# Editorial: Silicon: A “Quasi-Essential” element’s role in plant physiology and development

**DOI:** 10.3389/fpls.2023.1157185

**Published:** 2023-03-15

**Authors:** Abinaya Manivannan, Prabhakaran Soundararajan, Byoung Ryong Jeong

**Affiliations:** ^1^ National Institute of Plant Genome Research, Aruna Asaf Ali Marg, New Delhi, India; ^2^ Evolutionary Developmental (Evo-Devo) Genetics Laboratory, National Institute of Plant Genome Research, Aruna Asaf Ali Marg, New Delhi, India; ^3^ Department of Horticulture, Division of Applied Life Science (BK21 Four), Graduate School of Gyeongsang National University, Jinju, Republic of Korea; ^4^ Institute of Agriculture and Life Science, Gyeongsang National University, Jinju, Republic of Korea; ^5^ Research Institute of Life Science, Gyeongsang National University, Jinju, Republic of Korea

**Keywords:** silicon, crystal structure, nanosilicon, water deficit, drought stress

Silicon (Si) is the second most abundant element present in the Earth’s crust after oxygen, i.e., 28.8% in dry weight basis. Plants absorb Si in the form of orthosilicic acid [Si(OH)_4_]. Supplementation of Si has showed various beneficial effects on plants such as improved growth, yield, and tolerance against abiotic and biotic stress conditions. Owing to its benefits for plants, Si has been declared as a “Quasi-Essential” element. Accumulation of Si varies between plant species. Monocots such as rice accumulate approximately 10% of its dry weight, which is higher than essential elements such as nitrogen (N), phosphorus (P), and potassium (K). Application of Si has a remarkable impact against pathogens, pests, and insects invasion in several plant species (Song et al., 2021). Similarly, Si combats against various abiotic stresses such as drought, cold, salinity, UV-B, and heavy metals (Mir et al., 2022).

For the uptake of Si in *Oryza sativa*, low-silicon rice (Lsi1) acts as an influx transporter, and Lsi2 acts as an efflux transporter. Both are located in the distal and proximal end of the exodermis and endodermis of roots, respectively. Furthermore, OsLsi3 and OsLsi6 are involved in the xylem transportation and unloading of Si to the node and leaf tissues, respectively (Ma and Yamaji, 2015; Yamaji et al., 2015). Genes responsible for Si transportation have been identified in various plant species. However, there are no detailed reports about the structural aspects of Si transporters. The crystal structure of Lsi1 in both open (1.8 Å) and closed (3.0 Å) state have provided perspectives about silicic acid permeation and selective mechanism. Each monomer of Lsi1 consists of six transmembranes and two half-helixes connected by six loops forming a homotetramer. For the permeability of silicic acid, loop C and the selective filter (SF) interact by hydrogen bonding with Gly155-Arg222, Thr157-Thr223, and Val160-Ile213. Quantum mechanical/molecular mechanical (QM/MM) calculations have shown the possibility of interaction of the additional water molecule (Wat17) with Thr181 in addition to two unique water molecules (Wat3 and Wat9) interacting with Thr65 of the SF extended from the TM1. These three unique water molecules could limit silicic acid permeation in Lsi1 (Saitoh and Suga). Nevertheless, additional X-ray crystallography and NMR-related studies are required to be carried out between plants with a wide range of Si accumulation in order to elucidate the structural and functional differentiation of Si transporters.

Recent studies have shown that Si nanoparticles (nSi) have more advantages than orthosilicic acid due to their lesser size, higher surface-to-volume ratio, and maximum uptake or distribution (Verma et al.). Peng et al. unveiled the utilization of nSi to mitigate postharvest deterioration in ginger rhizomes and provide resistance against *Fusarium solani*. Addition of nSi enhanced the firmness of the rhizomes, reduced decay loss, and prevented oxidative stress. Particularly, nSi in a 100 mg L^−1^ concentration influenced the antioxidant mechanism and expression of genes involved in lignin synthesis and tolerance against *F. solani* in ginger. This illustrates that Si can also be considered as a potential alternative for the postharvest management of vegetables.

Though most horticultural crops are Si passive/non-accumulators, Si supplementation treatments have still shown enhanced tolerance against various stresses. The improvement of resistance has been proved by the increased activities of antioxidant enzymes that scavenge the excessively generated reactive oxygen species (ROS) in Si treatments. The association of Si with higher tolerance has been studied using proteomics approaches, and it has been reported that Si might be involved in the protection of various mechanisms such as photosynthesis, energy metabolism, redox homeostasis, transcription/translation, and hormone/cell signaling under salinity and hyperhydricity conditions (Soundararajan et al., 2017a; Soundararajan et al., 2017b). Most of the studies have been carried out either in hydroponics or *in vitro* conditions. Teixeira et al. conducted water-deficient experiments on sugarcane and energy cane with Si applied in intermediate and longer periods after transplanting. Interestingly, supplementation of Si showed a significant effect on growth promotion and an improved stoichiometry ratio of carbon:nitrogen:phosphorus, biomass, and water-use efficiency (WUE) during severe long-period water deficit conditions in both sugarcane and energy cane. Meanwhile, a field-based rain exclusion shelters experiment conducted in wheat suggested that Si significantly improved the biomass of wheat to 73% in drought condition and 43% in normal condition. Similarly, WUE was increased to 32-74% depending on the Si supplementation rate. Most importantly, the carbon content increased from 6.4 to 8.9 tons C ha^-1^ and 4.03 to 7.35 tons C ha^-1^ in normal and drought conditions, respectively. The Si treatments restored grain production in plants grown under drought conditions in amounts comparable to those grown under ambient rainfall conditions (Johnson et al.). It was predicted that in the future this biosphere will experience heavy water scarcity, which will affect our entire ecosystem. Studies on the application of Si and its transporters are much in need of climate-resilient crop development programs. To overcome drought and famine, in collaboration with agriculturalists, scientists need to explore sustainable strategies such as the utilization of Si fertilizers for food protection and ecological balance by considering its benefits (**Figure 1**).

**Figure 1:**
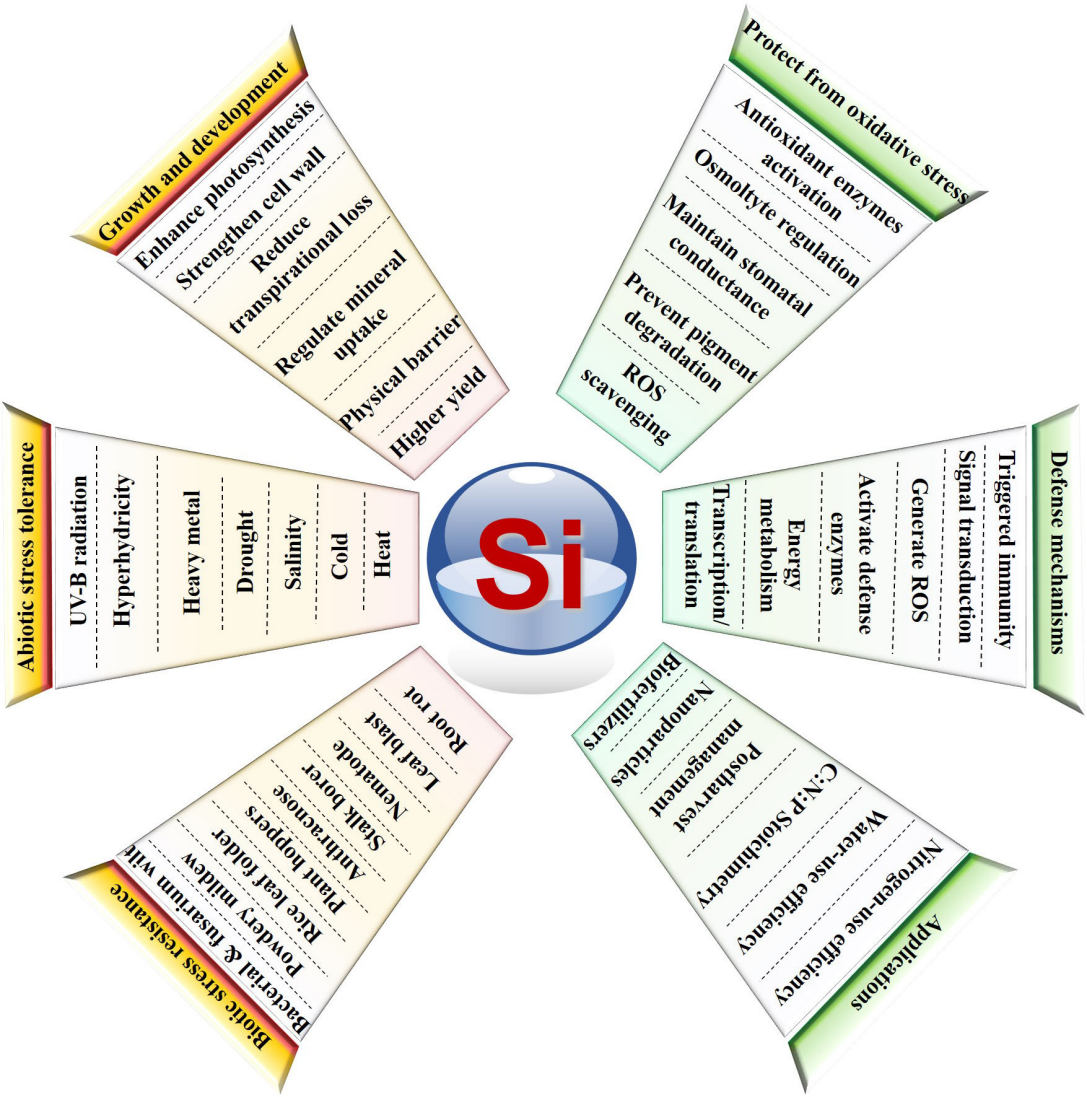
Beneficial effects of silicon (Si) in plant growth and development: overcoming abiotic stress and preventing pathogens and pests, as well as the key metabolic pathways it is involved with and its possible applications in the agriculture sector. Overall, this Research Topic covered the most important avenues, such as the structural mechanisms of silicic acid uptake, the advantages of nSi, the application of Si at the field level for its possible utilization in the sugarcane and energy cane industries, and wheat cultivation under drought conditions. In the changing climate conditions and with the proven results, Si can be used to eradicate pathogen invasion and as support for adaptive strategies in agri-sectors to increase profit margins for farmers as an environmentally friendly alternative.

## Author contributions

PS and AM prepared the manuscript. BJ reviewed and gave suggestions to the manuscript. All authors contributed to the article and approved the submitted version.
